# NeoProspect HER2-low: Response and Prognosis After Neoadjuvant Treatment in Patients with HER2-low Breast Cancer

**DOI:** 10.7759/cureus.103076

**Published:** 2026-02-06

**Authors:** Laura Pratas Guerra, Rafael Sá e Silva, Joana Simões, Margarida Quinto Pereira, Lisa Gonçalves, Marisa Couto, Maria Beatriz Gonçalves, Sofia Neves, Miguel Martins Braga, João Queirós Coelho, Maria João Sousa, Raquel Romão, Fernando Gonçalves, António Araújo

**Affiliations:** 1 Medical Oncology, Unidade Local de Saúde de Santo António, Porto, PRT; 2 Nuclear Medicine, Unidade Local de Saúde de Santo António, Porto, PRT; 3 Medical Oncology, Instituto Português de Oncologia Lisboa Francisco Gentil, Lisbon, PRT; 4 Medical Oncology, Unidade Local de Saúde de Santa Maria, Lisbon, PRT; 5 Medical Oncology, Unidade Local de Saúde São João, Porto, PRT; 6 Medical Oncology, Unidade Local de Saúde Trás-os-Montes e Alto Douro, Vila Real, PRT; 7 Pathology, Unidade Local de Saúde de Santo António, Porto, PRT

**Keywords:** breast cancer, breast neoplasms, human epidermal growth factor receptor 2, neoadjuvant therapy, pathological complete response, prospective studies, receptor erbb-2

## Abstract

Background: The majority of breast cancers (BCs) are classified as human epidermal growth factor receptor 2 (HER2)-negative. Within this group, more than half show low levels of HER2 expression, defined as an immunohistochemistry (IHC) score of 1+ or 2+ with a negative result on reflex in situ hybridization. The clinical and biological relevance of this HER2-low category remains under investigation, particularly regarding response to systemic therapy.

Methods: A national, multicentric, prospective, and observational study was conducted to evaluate the value of HER2-low as a predictive marker of pathological complete response (pCR) to neoadjuvant treatment in BC. Patients with HER2-low or HER2-zero (IHC 0), diagnosed at clinical stage I-III, were identified and followed throughout neoadjuvant treatment, consisting of chemotherapy ± immune checkpoint inhibitors, and subsequent surgery. Differences in subgroups and pCR were assessed using appropriate statistical tests.

Results: A total of 128 patients were included, of which 46% were HER2-low and 54% were HER2-zero, with a median age of 54 years and a median follow-up of 12 months. Within the HER2-low group, 68% patients were HER2-low 1+ and 32% were HER2-low 2+. In the HER2-low group, 71% of tumours were hormone receptor (HR) positive, and in the HER2-zero group, 67% were triple negative (TN). Tumours in the HER2-low group were more often HR-positive, postmenopausal, and had lower Ki67, while tumours in the HER2-zero group were more frequently TN, grade 3, and node-positive. Immunotherapy use was lower in HER2-low patients. Overall pCR rates were 20% in HER2-low, and 32% in HER2-zero (p=0.14), and pCR rates were lower in HR-positive compared with HR-negative tumours (12% vs. 41%). Among HER2-zero tumours, pCR was significantly lower in HR-positive than in HR-negative (9% vs. 43%; p=0.005). In HER2-low HR-positive, pCR was also lower than in the HER2-low HR-negative (14% vs. 35%; p=0.085). Within HER2-low, 23% of HER2 1+ and 16% of HER2 2+ obtained pCR. Pathological nodal response was more frequent in HER2-zero tumours (70% vs. 39%, p=0.009). Treatment was generally well tolerated, with grade ≥3 haematological toxicity being the most frequent one, without differences between the two groups.

Conclusions: This prospective real-world study suggests a trend toward lower pCR in HER2-low compared with HER2-zero tumours, although not statistically significant. The lower use of immunotherapy among HER2-low patients may have influenced these results and should be considered when interpreting comparative efficacy. The significantly reduced pCR in HR-positive compared with HR-negative disease within HER2-low tumours underscores the influence of HR status and highlights the need for careful pre-therapeutic stratification and larger, long-term studies.

## Introduction

Breast cancer (BC) is the most frequent cancer diagnosed in women worldwide, with approximately 2.3 million new cases reported globally in 2022 [1]. It represents a heterogeneous disease characterized by multiple molecular subtypes and clinicopathological features that influence treatment response and prognosis [2,3]. Human epidermal growth factor receptor 2 (HER2) is an oncogene and a well-established therapeutic target with HER2-directed therapies achieving substantial clinical benefit and high response rates not only in BC but also in other tumour types [2-4]. Approximately 20% of all BCs are classified as HER2-positive, characterized by HER2 protein overexpression or gene amplification, while the remaining 80% are considered HER2-negative [2].

However, within the HER2-negative group, increasing evidence highlights the relevance of the HER2-low category, defined by an immunohistochemistry (IHC) score of 1+ or 2+ with negative reflex in situ hybridisation (ISH) testing [5,6]. According to recent studies, HER2-low disease accounts for nearly 45-60% of all BC [7,8], underscoring its frequency in clinical practice. HER2-low tumours can be further divided into hormone receptor (HR)-positive, known as luminal, or triple-negative (TN) disease, depending on their HR expression profile [9,10].

The biological significance of HER2-low BC remains under debate. Some studies, including preliminary data, suggest that HER2-low tumours may represent a distinct biological entity, with different genomic and molecular profiles compared to HER2-zero tumours [11-13]. For instance, gene expression analyses have identified differences in immune-related pathways and tumour microenvironment composition, raising the possibility that HER2-low status influences tumour biology beyond serving as a predictive biomarker [7,12]. Conversely, other investigations failed to demonstrate meaningful molecular distinctions, supporting the view that HER2-low may represent a continuum within HER2-negative disease rather than a unique subtype [7,14-16].

The prognostic impact of HER2-low status remains inconsistent across studies. Some reports suggest slightly improved outcomes, particularly in HR-positive disease [7,17,18], whereas others show no significant survival differences [16,19,20]. In TN tumours, recent studies have highlighted clinical heterogeneity, with HER2-low TNBC displaying slightly better prognosis than HER2-zero TNBC in some cohorts [9,10,21-23], but not in others [24]. These discrepancies underscore the need for large, prospective studies to clarify the impact of HER2-low status on outcomes.

The predictive role of HER2-low status in response to neoadjuvant chemotherapy has also been investigated. Some studies indicate that HER2-low tumours may have lower rates of pathological complete response (pCR) compared to HER2-zero tumours [7,16-19], while a recent meta-analysis found no consistent association between HER2-low status and pCR rates across early BC populations [16]. This uncertainty emphasizes the necessity of further prospective, real-world data to inform clinical decision-making.

Antibody-drug conjugates (ADCs), particularly trastuzumab deruxtecan (T-DXd), have demonstrated robust activity in previously treated HER2-low advanced BC, significantly improving response rates and survival compared with standard chemotherapy [5,25]. These results have reshaped the treatment landscape and prompted investigation of T-DXd in earlier disease stages. Preliminary findings from trials such as DESTINY-Breast05 and DESTINY-Breast11 have shown encouraging improvements in invasive disease-free survival (I-DFS) and pCR, respectively, in high-risk early HER2-positive BC [25,26]. Early-phase neoadjuvant studies, such as TRIO-US B-12 TALENT (NCT04553770), have reported encouraging objective response rates in patients with HER2-low, HR-positive early BC, supporting the biological relevance of this subgroup and stimulating further trials in curative settings.

Taken together, the clinical, biological, and therapeutic relevance of HER2-low BC remains an evolving and highly debated area. With HER2-low tumours accounting for more than half of all BC cases, a deeper understanding of their prognosis and treatment response is essential to guide therapeutic strategies and optimize outcomes. The present study contributes to this growing body of evidence by evaluating neoadjuvant treatment response and prognostic features in patients with HER2-low BC within a large, prospective, multicentre cohort.

## Materials and methods

This was a national, multicentre, prospective, observational study carried out between January 2024 and August 2025. Patients were prospectively enrolled through multidisciplinary tumour boards when proposed for neoadjuvant treatment. All patients were informed about the study and provided written informed consent prior to participation. The study involved five centres: three university hospitals (Unidade Local de Saúde de Santo António and Unidade Local de Saúde de São João in Porto, and Unidade Local de Saúde de Santa Maria in Lisbon), one cancer centre (Instituto Português de Oncologia de Lisboa in Lisbon), and one local hospital (Unidade Local de Saúde Trás-os-Montes e Alto Douro in Vila Real) in Portugal. The study was approved by the Ethics Committee of Unidade Local de Saúde de Santo António e do Instituto de Ciências Biomédicas Abel Salazar (2023.121(104-DEFI/096-CE)). All hospitals had approval from their local ethics committees.

Study population

Eligibility Criteria

The inclusion criteria were the following: age ≥18 years; histologically proven invasive carcinoma of the breast; HER2-low or HER2-zero score assessed in the diagnostic biopsy; clinical stage I-III [27]; treatment with neoadjuvant chemotherapy ± immune checkpoint inhibitors followed by surgery. Exclusion criteria included HER2-positive status either in the biopsy or in the surgical specimen. A control group included patients with HER2-zero invasive BC, stages I-III [27], who also received neoadjuvant chemotherapy ± immune checkpoint inhibitors, followed by surgery.

Sample Size

This study was designed as a prospective, exploratory, observational analysis. Therefore, no formal sample size calculation was performed to detect a predefined difference in pCR rates between HER2-low and HER2-zero groups. The planned sample size was based on feasibility, expected patient accrual across participating centres, and the anticipated proportion of HER2-low tumours among HER2-negative breast cancers. A total sample of approximately 120-130 patients was considered sufficient to provide an initial estimation of pCR rates in both groups and to explore clinically meaningful trends that could inform the design of future, adequately powered studies.

Objectives

The primary objective was to evaluate the rate of pCR to neoadjuvant treatment in HER2-low BC and to compare it with HER2-zero disease. Pathological complete response was defined as the absence of all invasive cancer in both the breast and lymph nodes, irrespective of ductal carcinoma in situ, following completion of neoadjuvant treatment (ypT0/is ypN0) [28].

The secondary objectives included: (i) comparing biological and clinicopathological features between HER2-low and HER2-zero groups; (ii) evaluating event-free survival (EFS), defined as time to disease progression precluding surgery, local or distant recurrence, or death from any cause; (iii) distant disease-free survival (D-DFS), defined as time to metastasis or death; (iv) I-DFS, defined as time to earliest loco-regional or distant recurrence, invasive contralateral cancer, second primary cancer, or death; and (v) OS, defined as time from diagnosis to death from any cause [29]. The cut-off date for survival analysis was August 31, 2025; however, no survival results are presented due to the short follow-up period.

Definitions

HER2-low and HER2-zero classifications were determined according to the American Society of Clinical Oncology/College of American Pathologists (ASCO/CAP) guidelines [5,6]: HER2-low was defined as an IHC score of 1+, characterized by faint or barely perceptible incomplete membrane staining in >10% of tumor cells, or 2+ with negative ISH results; HER2-zero was defined as IHC 0, corresponding to the absence of membrane staining or faint/barely perceptible membrane staining in ≤10% of tumor cells. HER2 and HR testing, including Ki-67 assessment, were performed in local pathology laboratories, and additional clinicopathological variables were collected as detailed in the Results section.

The "NeoProspect cohort" is a prospective cohort of patients with BC treated in the neoadjuvant setting at the five participating Portuguese institutions; the present analysis focuses on the predefined subgroup of patients with HER2-low disease, with HER2-zero tumors analyzed as a comparator group.

Interventions

Germline testing was performed using a next-generation sequencing panel based on whole-exome sequencing for hereditary breast and ovarian cancer. This analysis included sequencing and copy number variant analysis of 20 susceptibility genes (*ATM, BARD1, BRCA1, BRCA2, BRIP1, CDH1, CHEK2, CTNNA1, EPCAM, MLH1, MSH2, MSH6, NBN, PALB2, PMS2, PTEN, RAD51C, RAD51D*, *STK11*, and *TP53*), as well as screening for the BRCA2 c.156_157insAlu Portuguese founder pathogenic variant.

Treatment regimens were determined by the treating physician and included: anthracycline dose-dense followed by taxane; carboplatin and taxane followed by anthracycline dose-dense; immunotherapy consisted of pembrolizumab, applicable in TN BC patients.

Calculations

The Residual Cancer Burden (RCB) score was calculated using the MD Anderson online calculator [30], which incorporates five pathological parameters: the two greatest dimensions of the primary tumour bed, percentage of residual invasive cancer cellularity, percentage of in situ disease within the tumour bed, number of positive lymph nodes, and the diameter of the largest nodal metastasis. RCB 0 corresponds to no residual disease, RCB-I to minimal, RCB-II to moderate, and RCB-III to extensive residual disease [30].

Statistical analysis

Statistical analysis was performed using IBM SPSS Statistics for Windows, version 27 (IBM Corp., Armonk, New York, United States). Descriptive statistics were used to summarize clinicopathological variables. Continuous variables were summarized as median with interquartile range and compared using the Mann-Whitney U test when distributions were non-normal, or the independent samples t-test when normality was verified using the Shapiro-Wilk test. Categorical variables were compared using the chi-square test or Fisher’s exact test, as appropriate. All tests were two-sided, with p < 0.05 considered statistically significant. No adjustments were made for multiple comparisons, given the exploratory nature of the analysis. 

## Results

A total of 128 patients were included, with 46% being HER2-low (n=59) and 54% HER2-zero (n=69), with a median follow-up time of 12 months. When HER2-low tumours were further stratified by IHC score, 40 patients were HER2-low 1+ and 19 were HER2-low 2+. The median age at diagnosis was 54 years, with HER2-low patients being slightly older than HER2-zero patients (55 vs. 49; p=0.016). All patients were female, and most had an Eastern Cooperative Oncology Group (ECOG) performance status (PS) of 0 (86%; n=110), and the remaining 14% an ECOG PS of 1 (n=18). 

Premenopausal status was more common in the HER2-zero group (59%) compared with the HER2-low group (32%; p=0.002). Tumour size at diagnosis did not differ significantly between HER2-low and HER2-zero groups (p=0.332). HER2-zero tumours were associated with higher clinical nodal stage (p=0.004) and were more frequently grade 3 (77% vs. 42%; p<0.001). HR positivity was higher in HER2-low tumours than in HER2-zero tumours (oestrogen receptors 71% vs. 33%; progesterone receptors 53% vs. 23%, respectively; both p<0.001), and the proportion of tumours with Ki67 >20% was significantly higher in the HER2-zero group compared with the HER2-low group (87% vs 73%; p=0.036). Within the HER2-zero group, 67% of tumours were TN, whereas HER2-low tumours were predominantly luminal B (63%). Ductal carcinoma was the most common histologic subtype (84%; n=108), followed by lobular (8%; n=10). Most patients were clinical stage II (62%; n=80) or III (32%; n=41). Among the 99 patients who underwent genetic germline testing (77%), four carried *BRCA1* pathogenic variants, six carried *BRCA2* pathogenic variants, two harboured other pathogenic germline alterations (*RAD51C* and *RAD51D*), and the remaining 87 were negative for germline pathogenic or likely pathogenic variants.

The baseline characteristics of both groups are detailed in Table 1.

**Table 1:** Baseline characteristics of HER2-low and HER2-zero patients. N/A: not applicable; ECOG: Eastern Cooperative Oncology Group; N: node; T: tumor; HER2: human epidermal growth factor receptor 2

Characteristics		Total (n=128)	HER2-zero (n=69)	HER2-low (n=59)	p value		Statistical test				Statistic value
Median age in years		54	49	55	0.016		Independent samples t-test				t = 2.43
Female sex, n (%)		128 (100)	69 (100)	59 (100)	N/A						
Baseline ECOG performance status, n (%)	0	110 (85.9)	62 (89.9)	48 (81.4)	0.168		Chi-square test				χ² = 1.90
	1	18 (14.1)	7 (10.1)	11 (18.6)							
Menopause status, n (%)	Pre/Perimenopausal	60 (46.9)	41 (59.4)	19 (32.2)	0.002		Chi-square test				χ² = 9.46
	Postmenopausal	68 (53.1)	28 (40.6)	40 (67.8)							
Median tumour size, mm		33	31	35	0.332		Mann–Whitney U test				N/A
Clinical T stage, n (%)	T1	19 (14.8)	13 (18.8)	6 (10.2)	0.477		Chi-square test				χ² = 2.49
	T2	73 (57.0)	39 (56.5)	34 (57.6)							
	T3	24 (18.8)	12 (17.4)	12 (20.3)							
	T4	12 (9.4)	5 (7.3)	7 (11.9)							
Clinical N stage, n (%)	N0	56 (43.8)	39 (56.5)	17 (28.8)	0.004		Fisher’s exact test				N/A
	N1	50 (39.0)	23 (33.3)	27 (45.8)							
	N2	17 (13.3)	4 (5.8)	13 (22.0)							
	N3	5 (3.9)	3 (4.4)	2 (3.4)							
Clinical stage, n (%)	IA	7 (5.5)	6 (8.7)	1 (1.7)	0.123		Fisher’s exact test				N/A
	IIA	53 (41.4)	31 (44.9)	22 (37.3)							
	IIB	27 (21.1)	15 (21.7)	12 (20.3)							
	IIIA	26 (20.3)	12 (17.4)	14 (23.7)							
	IIIB	10 (7.8)	2 (2.9)	8 (13.6)							
	IIIC	5 (3.9)	3 (4.4)	2 (3.4)							
Germline pathogenic variants, n (%) (n=99)	BRCA1	4 (4.0)	3 (5.0)	1 (2.6)	0.003		Fisher’s exact test				N/A
	BRCA2	6 (6.1)	1 (1.6)	5 (13.2)							
	Other	2 (2.0)	1 (1.6)	1 (2.6)							
	Not found	87 (87.9)	56 (91.8)	31 (81.6)							
Histoprognostic Factors Assessed in the Biopsy											
Histology subtype, n (%)	Ductal/non-special type	108 (84.4)	62 (89.9)	46 (78.0)	0.073		Fisher’s exact test				N/A
	Lobular	10 (7.8)	2 (2.9)	8 (13.6)							
	Mixed/Other	10 (7.8)	5 (7.2)	5 (8.4)							
Grade, n (%)	1	3 (2.3)	1 (1.5)	2 (3.4)	<0.001		Fisher’s exact test				N/A
	2	47 (36.7)	15 (21.7)	32 (54.2)							
	3	78 (61.0)	53 (76.8)	25 (42.4)							
Hormonal receptors (nuclear positivity), n (%)	Oestrogen receptor-positive	65 (50.8)	23 (33.3)	42 (71.2)	<0.001		Chi-square test				χ² = 18.23
	Progesterone receptor-positive	47 (36.7)	16 (23.2)	31 (52.5)	<0.001		Chi-square test				χ² = 11.79
Median Ki67		69	80	40	0.036		Mann–Whitney U test				N/A
Ki67 ≤20, n (%)		27 (21.1)	9 (13.0)	16 (27.1)	0.045		Chi-square test				χ² = 4.01
Ki67 > 20, n (%)		101 (78.9)	60 (87.0)	43 (72.9)							
Tumour subtype, n (%)	Luminal A	8 (6.3)	3 (4.3)	5 (8.5)	<0.001		Fisher’s exact test				N/A
	Luminal B	57 (44.5)	20 (29.0)	37 (62.7)							
	Triple negative	63 (49.2)	46 (66.7)	17 (28.8)							

Regarding neoadjuvant treatment, all patients in both groups received chemotherapy with a taxane (paclitaxel or docetaxel, 100%; n=128), the majority of patients received dose-dense anthracycline (97%; n=124), and less than half received carboplatin (48%, n=61) and pembrolizumab (43%; n=55). Pembrolizumab was administered significantly more often in HER2-zero than HER2-low patients (59% vs. 24%; p<0.001), as was carboplatin (67% vs. 25%; p<0.001). Grade ≥3 toxicity occurred in 21% of patients, the vast majority being haematological toxicity (neutropenia, anaemia, and thrombocytopenia), without significant differences between the two groups. Regarding immune-related toxicities, three cases of nephritis (two grade 3 and one grade 2), one case of grade 2 hepatitis, and one case of grade 2 dermatologic toxicity were registered. 

Imaging response, assessed by magnetic resonance imaging (MRI), showed complete response in 28 patients (22%), partial response in 88 (69%), stable disease in six (5%), and progression of disease in four (3%), with no significant differences between HER2-low and HER2-zero patients (p=0.162). Surgical approaches were comparable between groups, though axillary lymph node dissection was more frequent among HER2-low patients (64% vs. 45%; p=0.028).

Pathological assessment revealed smaller residual tumour size post-treatment in HER2-zero patients (6 vs. 20; p=0.033) and fewer lymph nodes with metastasis in these patients when compared to HER2-low (0 vs. 1; p<0.001). Pathological nodal negativity (ypN0) was higher in HER2-zero patients (70% vs. 39%; p=0.009). Overall, pathological TNM stage distribution differed significantly between groups (p=0.009), with HER2-zero tumours showing predominantly earlier-stage disease.

HER2-low group achieved lower pCR when compared to HER2-zero (20% vs. 32%; p=0.14), although it was not considered statistically significant. Pathological complete response rates were higher in the HER2-low 1+ group (n=9; 23%) compared with the HER2-low 2+ group (n=3; 16%), although this difference was not statistically significant (p=0.55). Similarly, RCB scores were numerically higher in HER2-low 2+ tumours compared with HER2-low 1+ tumours (2.83 vs. 1.42; p=0.068), suggesting a trend toward poorer treatment response with increasing HER2 expression intensity.

In the stratified analysis, the pCR rate was lower in HR-positive HER2-low tumours compared with HR-negative HER2-low tumours (14% vs. 35%; p=0.085). Among HER2-zero tumours, pCR rates were also lower in the HR-positive group (9% vs. 43%; p=0.005). These findings indicate that the detrimental effect of HR positivity on pCR appears slightly stronger within the HER2-low subgroup.

Treatment characteristics, pathological stage distribution, and overall therapeutic outcomes are summarised in Table 2, while the stratified pCR analyses are presented in Table 3.

**Table 2:** Treatment modalities, pathologic stage distribution, and therapeutic outcomes in HER2-low and HER2-zero breast cancer patients. N/A: not applicable; RCB: Residual Cancer Burden; TNM: tumor, node, metastatis; HER2: human epidermal growth factor receptor 2

		All (n=128)	HER2-zero (n=69)	HER2-low (n=59)	p value	Statistical test	Statistic value
Neoadjuvant treatment							
Chemotherapy regimen, n (%)	Taxane	128 (100)	69 (100)	59 (100)	N/A		
	Anthracycline	124 (96.9)	67 (97.1)	57 (96.6)	0.873	Chi-square test	χ² = 0.03
	Carboplatin	61 (47.7)	46 (66.7)	15 (25.4)	<0.001	Chi-square test	χ² = 20.07
Neoadjuvant pembrolizumab, n (%)		55 (43.0)	41 (59.4)	14 (23.7)	<0.001	Chi-square test	χ² = 15.11
Grade ≥ 3 toxicity to chemotherapy ± immune checkpoint inhibitors, n (%)		27 (21.1)	16 (23.2)	11 (18.6)	0.530	Chi-square test	χ² = 0.17
Imaging response							
Evaluation of response by MRI, n (%) (n=126)	Complete Response	28 (22.2)	19 (27.9)	9 (15.5)	0.162	Fisher’s exact test	N/A
	Partial Response	88 (69.8)	45 (66.2)	43 (74.2)			
	Stable Disease	6 (4.8)	3 (4.4)	1 (1.7)			
	Progression of Disease	4 (3.2)	1 (1.5)	5 (8.6)			
Surgery							
Type of breast surgery, n (%)	Unilateral mastectomy	52 (40.6)	26 (37.7)	26 (44.1)	0.764	Fisher’s exact test	N/A
	Bilateral mastectomy	7 (5.5)	4 (5.8)	3 (5.1)			
	Breast-conserving surgery	69 (53.9)	39 (56.5)	30 (50.8)			
Type of axillary surgery, n (%)	Sentinel lymph node biopsy	59 (46.1)	38 (55.1)	21 (35.6)	0.028	Chi-square test	χ² = 4.10
	Axillary lymph node dissection	69 (53.9)	31 (44.9)	38 (64.4)			
Histoprognostic factors assessed in the surgical specimen							
Median tumour size, mm		12	6	20	0.033	Mann–Whitney U test	N/A
Median number of lymph nodes with metastasis		0	0	1	<0.001	Mann–Whitney U test	N/A
Margin status, n (%)	R0	127 (99.2)	68 (98.6)	59 (100)	1.000	Fisher’s exact test	N/A
	R1	1 (0.8)	1 (1.4)	0 (0)			
Pathological TNM - T, n (%)	ypT0	32 (25.0)	18 (26.1)	14 (23.7)	0.187	Fisher’s exact test	N/A
	ypTis	6 (4.7)	5 (7.3)	1 (1.7)			
	ypT1mi	4 (3.1)	4 (5.8)	0 (0)			
	ypT1a	14 (10.9)	7 (10.1)	7 (11.9)			
	ypT1b	11 (8.6)	6 (8.7)	5 (8.4)			
	ypT1c	18 (14.1)	12 (17.4)	6 (10.2)			
	ypT2	28 (21.9)	12 (17.4)	16 (27.1)			
	ypT3	14 (10.9)	5 (7.2)	9 (15.3)			
	ypT4	1 (0.8)	0 (0)	1 (1.7)			
Pathological TNM - N, n (%)	ypN0	71 (55.5)	48 (69.6)	23 (37.7)	0.009	Fisher’s exact test	N/A
	ypN1a	30 (23.4)	12 (17.4)	18 (29.5)			
	ypN1mi	6 (4.7)	1 (1.4)	5 (8.2)			
	ypN2a	17 (13.3)	6 (8.7)	11 (18.0)			
	ypN3a	4 (3.1)	2 (2.9)	4 (6.6)			
Pathological staging TNM, n (%)	0	33 (25.8)	22 (31.9)	11 (18.6)	0.009	Fisher’s exact test	N/A
	IA	29 (22.7)	20 (29.0)	9 (15.3)			
	IB	17 (13.3)	9 (13.0)	8 (13.6)			
	IIA	21 (16.4)	5 (7.2)	16 (27.1)			
	IIB	8 (6.2)	6 (8.7)	2 (3.4)			
	IIIA	14 (10.9)	4 (5.8)	10 (16.9)			
	IIIB	5 (3.9)	2 (2.9)	3 (5.1)			
	IIIC	1 (0.8)	1 (1.5)	0 (0)			
Treatment response category, n (%)	Complete Response	34 (26.6)	22 (31.9)	12 (20.3)	0.250	Chi-square test	χ² = 2.77
	Partial Response	75 (58.6)	36 (52.2)	39 (66.1)			
	No response	19 (14.8)	11 (15.9)	8 (13.6)			
Median RCB calculator		1.55	1.35	1.96	0.068	Mann–Whitney U test	N/A
RCB Class, n (%)	0	34 (26.6)	22 (31.9)	12 (20.3)	0.442	Chi-square test	χ² = 2.69
	I	27 (21.1)	15 (21.7)	12 (20.3)			
	II	41 (32.0)	20 (29.0)	21 (35.6)			
	III	26 (20.3)	12 (17.4)	14 (23.8)			

**Table 3:** Distribution of pathologic complete response by HER2 and HR classification. N/A: not applicable; HER2: human epidermal growth factor receptor 2; HR: hormone receptor

	HER2-zero (n=69)		pvalue	Statistical test	Statistic value	HER2-low (n=59)		pvalue	Statistical test	Statistic value
	HR-positive	HR-negative				HR-positive	HR-negative			
Pathological complete response, n (%)	2 (8.7)	20 (43.5)	0.005	Fisher’s exact test	N/A	6 (14.3)	6 (35.3)	0.085	Fisher’s exact test	N/A
Without pathological complete response, n (%)	21 (91.3)	26 (56.5)				36 (85.7)	11 (64.7)			
Total, n (%)	23 (100)	46 (100)	N/A			42 (100)	17 (100)	N/A		

Treatment differences between groups, particularly the higher use of pembrolizumab and carboplatin in HER2-zero tumours, may partially account for their higher ypN0 and numerically superior pCR rates. This potential confounding effect should be considered when interpreting comparative efficacy outcomes.

Regarding adjuvant treatment, as expected, all HR-positive patients received endocrine therapy (n=69), most commonly with aromatase inhibitors, and in some cases combined with CDK4/6 inhibitors (n=14). Radiotherapy was delivered to 82% of patients (n=105), with similar distribution between HER2-low (81%) and HER2-zero (83%) groups.

## Discussion

In this prospective, multicentre, real-world cohort, HER2-low tumours accounted for almost half of all HER2-negative early BC cases, in line with contemporary estimates. Clinically, HER2-low disease was enriched for older, postmenopausal, HR-positive patients with lower grade and lower Ki67, whereas HER2-zero tumours more often displayed TN biology, higher grade, higher proliferative activity, and more advanced nodal stage at diagnosis. These findings support the view that HER2-low and HER2-zero occupy different regions of the HER2-negative spectrum, consistent with prior reports describing HER2-low as more frequently luminal-like and HER2-zero as more often fitting classical TNBC or highly proliferative profiles.

Imaging responses by MRI were similar between groups, but pathological outcomes revealed clinically meaningful differences. HER2-zero tumours showed a higher rate of nodal clearance (ypN0 70% vs. 39%) and a trend toward smaller residual primary tumour size and lower residual nodal burden, resulting in a more favourable post-treatment TNM stage distribution. Overall pCR was numerically higher in HER2-zero tumours (32% vs. 20% in HER2-low), although this difference did not reach statistical significance in our exploratory analysis. This pattern aligns with several retrospective series suggesting lower chemosensitivity in HER2-low compared with HER2-zero disease, particularly in cohorts enriched for HR-positive tumours [9,12,14,18], while also remaining consistent with findings from recent meta-analyses that did not demonstrate a uniform effect across all early BC populations [13,16,20].

Within HER2-low disease, stratification by IHC score (1+ vs. 2+) did not identify statistically significant differences in pCR or RCB class, but we observed a numerical trend toward higher pCR and lower RCB in HER2 1+ compared with HER2 2+ tumours. This finding is consistent with the concept of HER2 expression existing along a continuum rather than strictly separate entities and may suggest that increasing HER2 intensity within the HER2-low range is associated with slightly poorer chemotherapy response. However, given the modest sample size and known inter-observer variability in HER2 scoring, particularly at the 0/1+ boundary, these observations should be interpreted with caution and warrant confirmation in larger cohorts with central pathology review.

The most striking result of our study is the modifying effect of HR status on pCR within both HER2-low and HER2-zero tumours. Among HER2-low cancers, HR-positive tumours showed lower pCR rates than HR-negative tumours and a similar pattern was observed in the HER2-zero group. These findings highlight the adverse impact of HR positivity on chemosensitivity across the HER2-negative spectrum.

These data suggest that HER2-low status may interact with endocrine signalling and tumour biology to selectively attenuate chemosensitivity in HR-positive disease, while HR-negative HER2-low tumours behave more like classical TNBC in response to intensive chemotherapy ± immunotherapy. This is consistent with previous work showing lower proliferation and chemosensitivity in luminal HER2-low tumours and non-negligible biological heterogeneity within TNBC according to HER2 expression.

Interpretation of between-group differences must take into account treatment imbalances. Pembrolizumab and carboplatin were administered significantly more often in HER2-zero tumours, reflecting guideline-driven intensification in high-risk TNBC. The greater use of these agents likely contributed to higher nodal clearance and numerically higher pCR in the HER2-zero group and may partially explain the apparent disadvantage of HER2-low tumours, especially given their predominantly HR-positive profile. Nonetheless, toxicity was manageable overall, with grade ≥3 events in around one-fifth of patients and no significant safety differences between groups, supporting the feasibility of current neoadjuvant regimens in both HER2-low and HER2-zero disease in routine clinical practice.

Our findings are particularly relevant in the context of emerging HER2-targeting ADC for HER2-low disease. T-DXd has demonstrated substantial activity and survival benefit in HER2-low metastatic BC, and early neoadjuvant trials such as TRIO-US B-12 TALENT have reported promising objective responses in early HER2-low, HR-positive tumours [5,31]. Together with the modest pCR rates we observed in HR-positive HER2-low disease under standard chemotherapy, these data support the rationale for exploring ADC-based escalation strategies for patients with poor chemotherapy sensitivity, while future de-escalation strategies might be considered for subsets with excellent response. As evidence matures, HER2 expression level, in conjunction with HR status and other biomarkers, may become increasingly relevant for pre-therapeutic risk stratification and treatment selection in early HER2-negative BC.

This study has several strengths, including its prospective design, national multicentre real-world setting, and focus on contemporaneously treated HER2-low and HER2-zero cohorts, including patients receiving immune checkpoint inhibitors. Detailed pathological assessment with both pCR and RCB provides a nuanced view of residual disease beyond dichotomous endpoints. However, limitations must be acknowledged: the sample size is modest and not powered for definitive between-group comparisons; HER2 and HR status were assessed in local laboratories without central review; and no molecular or immune profiling was available to refine biological interpretation. Importantly, follow-up remains short (median 12 months), precluding robust assessment of EFS, D-DFS, I-DFS, or OS, and non-significant findings may reflect limited statistical power rather than true equivalence. The NeoProspect cohort remains under active follow-up, and survival analyses will be reported in future updates as data mature.

Despite these limitations, the NeoProspect cohort offers hypothesis-generating prospective evidence that HER2-low status is clinically and therapeutically relevant within HER2-negative early BC. HER2-low tumours are common and associated with distinct clinicopathological features; they show a trend toward lower pCR and less favourable nodal response than HER2-zero tumours in the context of less intensive systemic therapy; and HR-positive HER2-low disease appears particularly chemoresistant when compared with HR-positive HER2-zero tumours. Larger, centrally reviewed cohorts with integrated biomarker analyses and longer follow-up are warranted to clarify the prognostic impact of HER2-low status, optimize the use of ADCs and other targeted agents, and refine risk-adapted neoadjuvant strategies for this increasingly important subgroup.

## Conclusions

In this prospective multicentre cohort, HER2-low BC accounted for nearly half of HER2-negative cases and was more frequently associated with HR positivity, postmenopausal status, and lower Ki67 compared with HER2-zero disease. Although overall pCR rates did not differ significantly, a consistent trend toward lower pCR was observed in HER2-low tumours, particularly within the HR-positive subgroup, suggesting reduced chemosensitivity in this population. The NeoProspect cohort remains under active follow-up, and survival analyses will be reported in future updates as data mature.

These findings highlight the clinical relevance of HER2-low classification in early BC and support the need for careful biological stratification in treatment planning. Given the exploratory design and modest sample size, the results should be regarded as hypothesis-generating and require confirmation in larger studies with longer follow-up.
